# Accelerometer-Measured Daily Step Counts and Adiposity Indicators among Latin American Adults: A Multi-Country Study

**DOI:** 10.3390/ijerph18094641

**Published:** 2021-04-27

**Authors:** Gerson Ferrari, Adilson Marques, Tiago V. Barreira, Irina Kovalskys, Georgina Gómez, Attilio Rigotti, Lilia Yadira Cortés, Martha Cecilia Yépez García, Rossina G. Pareja, Marianella Herrera-Cuenca, Viviana Guajardo, Ana Carolina B. Leme, Juan Guzmán Habinger, Pedro Valdivia-Moral, Mónica Suárez-Reyes, Andreas Ihle, Elvio R. Gouveia, Mauro Fisberg, on behalf of the ELANS Study Group

**Affiliations:** 1 Escuela de Ciencias de la Actividad Física, el Deporte y la Salud, Universidad de Santiago de Chile (USACH), Santiago 7500618, Chile; gerson.demoraes@usach.cl (G.F.); monica.suarez@usach.cl (M.S.-R.); 2 CIPER, Faculdade de Motricidade Humana, Universidade de Lisboa, 1499-002 Lisbon, Portugal; adncmpt@gmail.com; 3 ISAMB, Faculdade de Medicina, Universidade de Lisboa, 1649-028 Lisbon, Portugal; 4 Department of Exercise Science, School of Education, University of Syracuse, Syracuse, NY 13210, USA; tvbarrei@syr.edu; 5 Carrera de Nutrición, Facultad de Ciencias Médicas, Pontificia Universidad Católica Argentina, Buenos Aires C1107AAZ, Argentina; ikovalskys@gmail.com; 6 Departamento de Bioquímica, Escuela de Medicina, Universidad de Costa Rica, San José 11501-2060, Costa Rica; georgina.gomez@ucr.ac.cr; 7 Centro de Nutrición Molecular y Enfermedades Crónicas, Departamento de Nutrición, Diabetes y Metabolismo, Escuela de Medicina, Pontificia Universidad Católica, Santiago 8330024, Chile; arigotti@med.puc.cl; 8 Departamento de Nutrición y Bioquímica, Pontificia Universidad Javeriana, Bogotá 110231, Colombia; ycortes@javeriana.edu.co; 9 Colegio de Ciencias de la Salud, Universidad San Francisco de Quito, Quito 17-1200-841, Ecuador; myepez@usfq.edu.ec; 10 Instituto de Investigación Nutricional, Lima 15026, Peru; rpareja@iin.sld.pe; 11 Centro de Estudios del Desarrollo, Universidad Central de Venezuela (CENDES-UCV)/Fundación Bengoa, Caracas 1053, Venezuela; manyma@gmail.com; 12 Nutrition, Health and Wellbeing Area, International Life Science Institute (ILSI) Argentina, Santa Fe Av. 1145, Caba C1059ABF, Argentina; viviana.guajardo@comunidad.ub.edu.ar; 13 Centro de Excelencia em Nutrição e Dificuldades Alimentaes (CENDA), Instituto Pensi, Fundação José Luiz Egydio Setubal, Hospital Infantil Sabará, São Paulo 01228-200, Brazil; acarol.leme@gmail.com (A.C.B.L.); mauro.fisberg@gmail.com (M.F.); 14 Family Relations and Applied Nutrition, University of Guelph, Guelph, ON N1G 2W1, Canada; 15 Department of Nutrition, School of Public Health, University of São Paulo, São Paulo 01246-904, Brazil; 16 Sports Medicine and Physical Activity Specialty, Science Faculty, Universidad Mayor, Santiago 8580745, Chile; juan.guzmanh@mayor.cl; 17 Faculty of Science Education, Campus de Cartuja, University of Granada, 18071 Granada, Spain; 18 Center for the Interdisciplinary Study of Gerontology and Vulnerability, University of Geneva, 1205 Geneva, Switzerland; andreas.ihle@unige.ch; 19 Swiss National Centre of Competence in Research LIVES—Overcoming Vulnerability: Life Course Perspectives, Lausanne and Geneva, 1022 Chavannes-près-Renens, Switzerland; 20 Department of Psychology, University of Geneva, 1205 Geneva, Switzerland; 21 Departamento de Educação Física e Desporto, Universidade da Madeira, 9020-105 Funchal, Portugal; erubiog@staff.uma.pt; 22 Interactive Technologies Institute, LARSyS, 9020-105 Funchal, Portugal; 23 Departamento de Pediatria, Universidade Federal de São Paulo, São Paulo 04023-061, Brazil

**Keywords:** physical activity, walking, accelerometer, moderate-to-vigorous physical activity, overweight, obesity, Latin America, epidemiologic study

## Abstract

The aim of the present study was to examine the sex-related associations between accelerometer-measured daily step counts and adiposity indicators in adults from eight Latin American countries. We analyzed data from 2524 adults (aged 18–65 years) from the Latin American Study of Nutrition and Health. Device-measured daily step counts were measured by accelerometers (ActiGraph GT3X). The outcomes were body mass index (BMI; (kg/m2), waist and neck circumference (in cm). Overall, the mean of daily steps counts, BMI, waist and neck circumference were 10699.8, 27.3, 89.6, and 35.8. Weak and negative associations were observed between daily steps counts and BMI (*r* = −0.17; *p* < 0.05) and waist circumference (*r* = −0.16; *p* < 0.05); however, step counts was not associated with neck circumference. Daily steps counts were negatively associated with BMI (β: −0.054; 95%CI: −0.077; −0.012) and waist circumference (−0.098; −0.165; −0.030) independently of age and socioeconomic level. In men, there were significant negative associations between daily steps counts with BMI (−0.075; −0.119; −0.031) and waist circumference (−0.140; −0.233; −0.048), and in women, there was no significant association with either of the body composition indicators. The findings from this study need to be examined in prospective settings that use device-measured from Latin America.

## 1. Introduction

The most recently reported prevalence of overweight and obesity in adults around the world was approximately 39%, which is the highest prevalence ever reported [1]. One of the most affected regions is Latin America, where alarming rates of up to 60% were reported [2]. This generates enormous public health costs in relation to morbidity and mortality. This is explained by the extent of the association between adiposity and the increased risk of diabetes mellitus, cardiovascular diseases, and various types of cancer [3]. These diseases correspond to the leading causes of death in Latin America and the world [4,5].

Excess adiposity is a tremendously complex problem, with multiple causes involving both environmental and individual factors [6]. One factor that is involved is physical inactivity [7]. Multiple barriers are responsible for individuals not achieving recommendations on physical activity for health. In Latin America, environmental factors that can inhibit physical activity performances include the unregulated expansions of urban environments, high-income inequality, high population density, population aging, air pollution, high crime rates, and high poverty levels [8,9,10]. These barriers must be well identified, and work in an interdisciplinary–intersectoral way to solve them. Thus, one way to mitigate the epidemic of overweight and obesity is by promoting healthy lifestyles, in which physical activity has a preponderant role [11]. Physical activity can be used to reduce the risk of obesity, lower the risk of all-cause mortality, cardiovascular events, and type 2 diabetes [12,13]. There are multiple types of physical activity, but the most common type in the world is walking [14]. Walking does not require special training and can be done almost everywhere [15].

Inconsistent methods can increase the proportion of errors and mask or alter the actual associations between physical activity and health outcomes [16]. Most studies from low- and middle-income countries use surveys where high levels of reporting bias can be found [16,17,18]. In the Latin America region, low correlations were established when contrasting with device-measured methods, such as accelerometers [16]. Although the importance of these data (in terms of device measures) are unquestionable, accelerometers are relatively expensive and require additional personnel time/expertise to manage and process the data to derive the endpoints. Accelerometers were used to capture/describe step data in nationally representative surveys from high-income countries [19]. On the other hand, device-measured methods are rare in countries from Latin America, where (most of the) previous researchers used indirect measures (i.e., questionnaires) to assess physical activity [20,21]. However, by using accelerometers, it is possible to accurately and reproducibly count daily steps [22].

Studies from high-income countries have suggested that daily step counts are associated with lower adiposity, such as body mass index (BMI) or waist circumference [23,24]. Daily step counts (as an indicator of overall physical activity volume) would help identify the strength of the association with the risk of overweight and obesity [20]. In regards to daily step counts—there is a lack of studies that can enhance precision (with a standard design and comparable methods) across Latin American countries. The Latin American Study of Nutrition and Health (Estudio Latinoamericano de Nutrición y Salud—ELANS) is a nationally representative data source that includes accelerometer-measured step counts and adiposity indicators. The availability of multi-center cross-sectional survey data from ELANS provides the rare opportunity to examine how accelerometer-measured daily step counts are associated with adiposity indicators in eight Latin American countries. Moreover, this will help with the development of public health policies and behavioral-change strategies to promote physical activity. The aim of the present study was to examine the sex-related associations between accelerometer-measured daily step counts and adiposity indicators in adults from eight Latin American countries.

## 2. Materials and Methods

### 2.1. ELANS Study Design and Participants

ELANS was a multi-national cross-sectional study that collected accelerometer-measured daily step counts and adiposity indicators across eight Latin American countries: Argentina, Brazil, Chile, Colombia, Costa Rica, Ecuador, Peru, and Venezuela [25,26]. ELANS was an epidemiological study using a standard design with comparable methods across countries. The study used a large representative sample from these eight countries and focused on urban populations. Only data for urban locations were included to increase comparability across countries and for reasons surrounding feasibility. Data were collected from September 2014 to February 2015. The overarching ELANS protocol was approved by the Western Institutional Review Board (#20140605) and although the study is not a clinical trial, the protocol was registered at ClinicalTrials.gov (#NCT02226627). Ethical approval was obtained from each local institutional review board and all participants provided written informed consent.

Sample size calculation considered a survey design effect of 1.75 with a limit error of 3.5% and *p* < 0.05, resulting in a required sample size of 9090, and was estimated based on guidance from the National Center for Health Statistics [27]. The study was conducted with a complex and multi-stage cluster-stratified sample design, with all regions for each country represented and a random selection of main cities in each region, according to the probability proportional to size. The sample was stratified by sex, age range (15–19.9, 20–34.9, 35–49.9, and 50–65 years), and socioeconomic level (low, middle, and high). Socioeconomic levels were weighted according to the national indices of each country. Details about participant sampling and recruitment strategies were published elsewhere [25,26].

Participants (15–65 years old) were recruited and the final sample included 9218 (4809 [51.9%] men) adolescents and adults. Daily step counts were collected for 40% of the sample, randomly selected to fill quotas by sex, age, and socioeconomic level, thereby ensuring a representative subsample across these dimensions. For logistical and financial reasons, efforts were made to ensure that a range of 23.4–34.2% of each sample wore the devices for seven consecutive days. The current manuscript is based on a sample of 2524 participants aged 18–65, with valid data for accelerometer-measured daily step counts, representing 27.4% of the total ELANS cohort. There were no significant differences (*p* > 0.05) in socioeconomic levels and distribution by sex between the participants who were asked to wear accelerometers and those who were not. We excluded participants ≤17 years of age from the analyses because the ELANS did not include adolescents <15 years of age, and this specific age (15–17 years) was not considered in the sample weight. Moreover, adolescents may have different physical activity behaviors when compared to adults [28]. Moreover, physical activity guidelines for adolescents differ than guidelines for adults [12].

The protocol used in this manuscript included data collected during two home visits. The first visit included an assessment of adiposity indicators. Additionally, a subsample of the designated respondents received instructions regarding the use of accelerometers to measure daily step counts; they were also provided with diaries (to complete for seven consecutive days). The second visit, which included retrieving the accelerometers and diaries, occurred 8 days after the first visit for participants who were provided with the accelerometers.

### 2.2. Device-Measured Daily Step

The GT3X+ accelerometers (ActiGraph, Pensacola, FL, USA), considered valid in adults [19,29], were used to measure daily step counts. Daily step counts measured over 7 days using the ActiGraph GT3X+ was previously shown to correlate (*r* = 0.86 for men; *r* = 0.89 for women) well to physical activity energy expenditure as measured by doubly labeled water [30]. The accelerometer was worn at the waist on an elasticized belt on the right mid-axillary line. At least 10 h/day for 7 days (including at least one day over the weekend) of recorded wear time were considered valid. Participants were asked to wear the devices while awake and remove them only for showering/bathing or other water activities and when sleeping.

The research team went to the participants’ homes to retrieve the devices on a subsequent day, of device data collection. The team downloaded the data using the latest version of the ActiLife software (version 6.0; ActiGraph, Pensacola, FL). Data were collected at a sampling rate of 30 Hz and processed to 60-s epochs [31].

After excluding the nighttime sleep period, non-wear waking time was defined by any sequence of ≥60 consecutive minutes of 0 activity counts [32]. The mean valid days of wear time and mean number of hours of daily wear time within the analyzed sample with five or more valid days was 6.6 (95% confidence interval [CI]: 6.2; 7.0) and 15.3 h per day (95%CI: 15.1; 15.5), respectively. Details were previously published [33].

To calculate mean daily step counts, the total number of steps during the valid wear period was divided by the number of valid days that each participant was wearing the accelerometer.

### 2.3. Adiposity Indicators

In each country, the adiposity indicators (body weight, height, waist, and neck circumferences) were measured while the participant wore light clothing and without shoes using standard procedures and equipment. Body height (to the nearest 0.1 cm) and body weight (to the nearest 0.1 kg) were measured using a calibrated electronic scale Seca 213^®^ (Hamburg, Germany). The measurements were taken during the end inhalation with the participant’s head in the Frankfort Plane, without shoes [34]. BMI was calculated as weight (kg) divided by height squared (m^2^).

A non-stretchable tape with an accuracy of 0.1 cm was used to evaluate circumferences. Waist circumference was measured according to World Health Organization recommendations, i.e., with the participants standing, after regular expiration, to the nearest cm, midway between the lowest rib and the iliac crest on the horizontal plane, with the subject in a standing position [35]. Neck circumference was measured in a plane as horizontal as possible, immediately below the laryngeal prominence, while standing erect with eyes facing forward [36]. All measurements were performed under strictly standardized conditions. Two measurements of the adiposity indicators were performed, and the mean was used for the analyses.

### 2.4. Statistical Analysis

The samples were weighted considering sociodemographic variables, sex, and socioeconomic level, to make the sample comparable with the whole population of each country [25]. Statistical analyses were carried out with the software SPSS v.26 software (SPSS Inc., IBM Corp., Armonk, New York, NY, USA) [37]. Means, standard deviations (SD), and percentages were computed, as appropriate, to describe the variables.

Differences between sexes were analyzed using a t-test for independent samples. A Pearson correlation coefficient (r) was used to assess the association of daily step counts with adiposity indicators (BMI, waist, and neck circumference). A coefficient value of <0.30 was considered as weak, 0.30 to 0.49 as moderate, and >0.50 as strong [38]. Multilevel linear regression models (β; confidence interval 95% [95%CI]) were used to examine the associations between daily step counts with adiposity indicators in adults. We adjusted the models for age and socioeconomic level. Statistical significance was considered at *p* < 0.05 and all tests were two-sided.

## 3. Results

The descriptive characteristics of the study sample are provided in Table 1, stratified by country. The total number of participants included in this study was 2524 (53.1% women) (aged 18.0–65.0 years). The mean (SD) age of the total sample was 38.3 (13.4) years, and the mean (SD) of daily step counts, BMI, waist, and neck circumference were 10699.8 (5148.6), 27.3 (5.4), 89.6 (13.8), and 35.8 (4.1), respectively. The mean daily step counts ranged from 7586.8 in Costa Rica to 15192.8 in Chile; the mean BMI ranged from 27.0 in Ecuador to 28.3 in Chile; the mean waist circumference ranged from 87.9 in Ecuador to 93.6 in Costa Rica, and the mean neck circumference ranged from 34.7 in Brazil to 37.4 in Chile.

Table 2 presents descriptive characteristics of men and women. Overall, men and women did not significantly differ in mean age and waist circumference; however, device–measured daily step counts and neck circumferences were significantly greater in men when compared to women. On the other hand, body mass index was significantly greater in women than in men.

Table 3 presents the results of the correlation analysis describing correlations between accelerometer-measured daily step counts and adiposity indicators (BMI, waist and neck circumference) variables in total and by sex. Weak and negative correlations were observed between device-measured daily steps counts, BMI, and waist circumference; however, daily step counts were not significantly correlated with neck circumference. In men, daily step counts were weakly and negatively correlated with each of the adiposity indicators. On the other hand, daily step counts were not correlated with either of the adiposity indicators in women.

Table 4 presents the results of the multilevel linear regression analysis describing the association between accelerometer-measured daily step counts and each adiposity indicator by sex adjusted for age and socioeconomic level. For men and women combined, daily step counts were negatively associated with body mass index and waist circumference. In men, there were significant negative associations between daily step counts with body mass index and waist circumference adjusted for age and socioeconomic level, and in women, there was no significant association with either of the adiposity indicators.

## 4. Discussion

The present study examined the sex-related associations between accelerometer-measured daily step counts and adiposity indicators among adults from eight Latin American countries. In general, daily step counts were negatively associated with BMI and waist circumference independently of age and socioeconomic level. However, when stratified by sex, a significant association was only observed among men among daily step counts and BMI, and waist circumference.

BMI and waist circumference are the two most common indicators of adiposity included in studies that analyze daily steps [39,40]. It was reported that people who accumulated more steps per day had a lower BMI and waist circumference [39,41], which is what was observed in the general sample, and in men. Similar to other findings [42,43], the associations observed in the current study between BMI and waist circumference were statistically significant in both sexes. Nevertheless, the linear regression models presented suggest that the preferred obesity index is different in men and women. While waist circumference, and not BMI, reflects fat distribution, neither waist circumference nor BMI measures body tissue composition. Men and women differ considerably in fat proportion, as well as distribution. Sex-related differences, which are readily apparent in normal-weight men and women, may predispose to a spectrum of fat distribution phenotypes with obesity [44]. Higher prevalence of “apple” shaped obesity in men, i.e., central obesity (“android obesity”) may explain the stronger relationship we observed in waist circumference in men. Sex-based differences in fat distribution may explain differences between the sexes in adiposity indicators.

Like our results, recent device-measured studies have also found that daily step counts are higher in men than women [45,46]. Additionally, a study from ELANS showed that men were more active at moderate-to-vigorous physical activity than women (42.1 min/day; 95%CI: 40.8; 43.4 versus 28.3 min/day; 95%CI: 27.1, 29.6) with women recording greater volumes of light activities (316.0 min/day; 95%CI: 311.3; 320.7 versus 305.7 min/day; 95% CI: 300.8; 310.7) [33]. An explanation for these confirmed sex differences was the maintenance of traditional gender roles, with men engaging in heavier activities and making more daily journeys as “wage-earners” of the family, while women were more involved in light domestic activities, such as home management, care, and cooking. Daily journey logs confirmed that those making most journeys away from their homes were more likely to be the more active men [47].

The negative correlation observed between steps per day and the indicators of adiposity in men, although statistically significant, was weak. Thus, the daily step counts explained only between 3 and 4% of the variance of the BMI and waist circumference, respectively. Therefore, the clinical application is limited. Based on the beta coefficients, an increase of 14,313 steps per day is associated with a decrease of 1 point in BMI; which, in practical terms, implies an increase of more than double the average number of steps reported in our sample. In contrast, an increase of 2813 steps per day was associated with a decrease of 1 cm in waist circumference. The decrease in waist circumference of 1 cm may be within the range of the measurement error, and it is more achievable than the increase in the number of daily steps associated with a decrease in BMI. Thus, the number of steps needed to achieve a relevant difference in BMI or waist circumference is challenging.

The low association observed between daily steps and adiposity in men, and the absence of associations observed in women, could be affected by other factors. The large amount of time in sedentary behavior previously reported in the Latin American population could at least partially explain our results [48,49,50]. Using data from the ELANS study, we showed there were only minor sex differences in the average levels of device-measured sedentary behavior (men: 582.7 min/day; 95%CI: 576.2; 589.2; women: 561.6 min/day; 95%CI: 555.3; 567.8) [33]. Previous research has shown that substituting 30 min of sedentary behavior to light physical activity may reduce the risk of all-cause mortality by 11% and risk of cardiovascular disease by 24% [51]. The recommendation to limit sedentary behavior was qualified with an acknowledgment that replacing sedentary time with any intensity of physical activity (including light intensity) has health benefits. This recommendation was based first on the juxtaposed evidence of lower levels of time spent in sedentary behaviors being beneficial for health, even among those with modest levels of moderate-to-vigorous physical activity [12]. The important practical application of this recommendation is to encourage the promotion of multiple approaches, in order to limit the adverse health outcomes associated with high levels of sedentary time. Considering the negative effects of sedentary behaviors on health outcomes [52], there is a need to monitor these behaviors in national health surveys. The importance of strategies aimed at reducing sedentary behavior should also be included in national policies. Currently, there are no strategies that aim to reduce sedentary behavior in South America.

Unlike BMI and waist circumference, neck circumference, despite being an indicator of adiposity [36], has been less considered in studies that analyzed daily steps. Previous research investigated its relationship with different types of physical activity. When using accelerometers, neck circumference is only negatively associated with vigorous physical activity in men and no association was observed with daily steps [53]. Similar to our results, these associations were not observed in women. The use of neck circumference was proposed as an alternative measure of adiposity; however, its association with body composition appears to be less strong than BMI or waist circumference [54]. Future research is needed to analyze the use of neck circumference in combination with BMI or waist circumference to estimate adiposity.

Some limitations must be considered. The accelerometers do not capture common activities, such as cycling, resistance, and static exercise, and carrying loads. Our evaluation of accelerometer-measured daily step counts may not represent the total population in the eight participating countries, since participants were recruited from specific urban neighborhoods. The most significant barriers to conducting studies such as this one are the inherent organizational complexities, recruitment of collaborators and research staff, institutional cooperation, development of infrastructure, and identification of resources. Multicenter cooperative studies, while challenging, offer great potential for building a scientific base for studies on physical activity and health. Importantly, the strength of this study concerns its accelerometer-measured daily step counts as a dimension of physical activity in a large sample size of the Latin American population. Accelerometers, such as those used in ELANS, are valid instruments to measure steps and physical activity intensity. The use of device methods is rare in Latin American countries where most previous research relied on self-reported instruments [33]. The use of the accelerometer is not influenced by recall biases, which is a common disadvantage of self-report methods. Combining an accelerometer and a previously validated questionnaire is of great interest, especially to better understand the reality in Latin America in regards to physical activity spaces and domains. Our study is the first to evaluate daily step counts via devices in Latin America, using a standardized methodology across a consortium of several participating countries.

## 5. Conclusions

This present study showed an inverse association between accelerometer-measured daily step counts and adiposity indicators, specifically in regards to BMI and waist circumference in men and women combined, and for men alone.

This analysis of accelerometry data and adiposity indicators represents the first examination of these associations in the Latin America region. This study demonstrates that total physical activity volume is an important factor for health, and efforts to improve daily step counts, due to the health benefits, should be undertaken. Further research is required to ascertain the more intricate relationship between daily step counts and adiposity indicators at different levels (i.e., lower or higher BMI) in Latin American adults.

## Figures and Tables

**Table 1:** Descriptive characteristics of the participants stratified by country.

Country	Men/Women ^a^	Age (years) ^b^	Daily Step Counts ^b^	Body Mass Index (kg/m^2^) ^b^	WC (cm) ^b^	NC (cm) ^b^
Argentina (*n* = 271)	42.1/57.9	40.7 (13.0)	7933.8 (3145.6)	28.0 (5.7)	91.2 (15.6)	36.2 (4.1)
Brazil (*n* = 524)	44.1/55.9	39.1 (13.3)	14120.0 (5181.2)	27.5 (5.6)	88.9 (13.9)	34.7 (4.6)
Chile (*n* = 274)	46.4/53.6	38.7 (13.2)	15192.8 (4669.7)	28.3 (5.2)	93.3 (12.5)	37.4 (3.7)
Colombia (*n* = 319)	49.8/50.2	39.5 (13.9)	8192.7 (3561.7)	25.6 (4.5)	85.6 (11.6)	35.2 (3.3)
Costa Rica (*n* = 247)	47.4/52.6	38.5 (12.8)	7586.8 (3498.3)	28.1 (6.2)	93.6 (16.4)	36.8 (3.9)
Ecuador (*n* = 249)	50.2/49.8	36.5 (13.6)	8336.3 (3505.4)	27.0 (5.2)	87.9 (11.4)	35.3 (3.6)
Peru (*n* = 302)	47.0/53.0	37.1 (13.4)	8521.2 (3612.8)	27.2 (4.9)	88.9 (12.3)	35.7 (3.7)
Venezuela (*n* = 338)	49.7/50.3	36.1 (13.2)	12285.3 (4983.3)	27.1 (5.6)	88.8 (14.5)	36.4 (4.4)
All countries (*n* = 2524)	46.9/53.1	38.3 (13.4)	10699.8 (5148.6)	27.3 (5.4)	89.6 (13.8)	35.8 (4.1)

^a^: Data are shown as a percentage; ^b^: data are shown as mean (standard deviation); abbreviations: WC: waist circumference; NC: neck circumference.

**Table 2:** Descriptive characteristics of participants stratified by sex.

	Men (*n* = 1183)	Women (*n* = 1341)	*p* ^a^
Age (years)	37.2 (13.5)	39.4 (13.2)	0.934
Daily step counts	11323.9 (5475.8)	10148.4 (4775.7)	<0.001
Body mass index (kg/m^2^)	26.7 (5.1)	27.9 (5.7)	<0.001
Waist circumference (cm)	91.0 (13.9)	88.4 (13.6)	0.410
Neck circumference (cm)	38.0 (3.8)	33.9 (3.3)	0.004

Data are shown as mean (standard deviation); ^a^ *p*-value for differences between sexes (*t*-test for independent samples).

**Table 3:** Bivariate correlations between accelerometer-measured daily step counts and adiposity indicators stratified by sex.

Sex	Body Mass Index (kg/m^2^)		Waist Circumference (cm)		Neck Circumference (cm)	
	*r*	*p*	*r*	*p*	*r*	*p*
Total	−0.17	<0.001 *	−0.16	0.005 *	0.11	0.783
Men	−0.18	0.012 *	−0.20	<0.001 *	−0.18	0.005 *
Women	−0.14	0.116	−0.14	0.157	−0.14	0.189

* Value of significance of the Pearson correlation coefficient.

**Table 4:** Multilevel linear regression models (β coefficient, 95%CI) between device-measured daily step and adiposity indicators stratified by sex.

Sex	Body Mass Index (kg/m^2^)		Waist Circumference (cm)		Neck Circumference (cm)	
	*β* (95%CI)	*p*	*β* (95%CI)	*p*	*β* (95%CI)	*p*
Total	−0.054 (−0.077; −0.012)	0.011	−0.098 (−0.165; −0.030)	0.007	−0.021 (−0.050; 0.020)	0.072
Men	−0.075 (−0.119; −0.031)	0.003	−0.140 (−0.233; −0.048)	0.006	−0.032 (−0.061; 0.031)	0.086
Women	−0.004 (−0.050; 0.057)	0.735	−0.020 (−0.131; 0.011)	0.634	−0.012 (−0.039; 0.029)	0.832

Abbreviations: CI: confidence interval; multilevel linear regression models, including region and cities as random effects adjustment for age and socioeconomic level.

## Data Availability

The datasets generated and/or analyzed during the current study are not publicly available due the terms of consent/assent to which the participants agreed, but are available from the corresponding author upon reasonable request. Please contact the corresponding author to discuss availability of data and materials.

## References

[1] World Health Organisation (WHO) Obesity and Overweight. https://www.who.int/en/news-room/fact-sheets/detail/obesity-and-overweight.

[2] Kovalskys I., Fisberg M., Gomez G., Pareja R.G., Yepez Garcia M.C., Cortes Sanabria L.Y., Herrera-Cuenca M., Rigotti A., Guajardo V., Zalcman Zimberg I. (2018). Energy intake and food sources of eight Latin American countries: Results from the Latin American Study of Nutrition and Health (ELANS). Public Health Nutr..

[3] Abdelaal M., Le Roux C.W., Docherty N.G. (2017). Morbidity and mortality associated with obesity. Ann. Transl. Med..

[4] Carioli G., Bertuccio P., Malvezzi M., Rodriguez T., Levi F., Boffetta P., La Vecchia C., Negri E. (2020). Cancer mortality predictions for 2019 in Latin America. Int. J. Cancer.

[5] Mortality G.B.D., Causes of Death C. (2015). Global, regional, and national age-sex specific all-cause and cause-specific mortality for 240 causes of death, 1990–2013: A systematic analysis for the Global Burden of Disease Study 2013. Lancet.

[6] Allender S., Owen B., Kuhlberg J., Lowe J., Nagorcka-Smith P., Whelan J., Bell C. (2015). A community based systems diagram of obesity causes. PLoS ONE.

[7] Booth F.W., Roberts C.K., Thyfault J.P., Ruegsegger G.N., Toedebusch R.G. (2017). Role of inactivity in chronic diseases: Evolutionary insight and pathophysiological mechanisms. Physiol. Rev..

[8] Salvo D., Reis R.S., Sarmiento O.L., Pratt M. (2014). Overcoming the challenges of conducting physical activity and built environment research in Latin America: IPEN Latin America. Prev. Med..

[9] Lund C., De Silva M., Plagerson S., Cooper S., Chisholm D., Das J., Knapp M., Patel V. (2011). Poverty and mental disorders: Breaking the cycle in low-income and middle-income countries. Lancet.

[10] United Nations (2012). World Urbanization Prospects: The 2011 Revision: Data Tables and Highlights 2011 Rev.

[11] Jakicic J.M., Davis K.K. (2011). Obesity and physical activity. Psychiatr. Clin. N. Am..

[12] Bull F.C., Al-Ansari S.S., Biddle S., Borodulin K., Buman M.P., Cardon G., Carty C., Chaput J.P., Chastin S., Chou R. (2020). World Health Organization 2020 guidelines on physical activity and sedentary behaviour. Br. J. Sports Med..

[13] Kraus W.E., Janz K.F., Powell K.E., Campbell W.W., Jakicic J.M., Troiano R.P., Sprow K., Torres A., Piercy K.L. (2019). Daily step counts for measuring physical activity exposure and its relation to health. Med. Sci. Sports Exerc..

[14] Malone S.K., Patterson F., Grunin L., Melkus G.D., Riegel B., Punjabi N., Yu G., Urbanek J., Crainiceanu C., Pack A. (2020). Habitual physical activity patterns in a nationally representative sample of U.S. adults. Transl. Behav. Med..

[15] Hansen B.H., Dalene K.E., Ekelund U., Wang Fagerland M., Kolle E., Steene-Johannessen J., Tarp J., Alfred Anderssen S. (2020). Step by step: Association of device-measured daily steps with all-cause mortality—A prospective cohort Study. Scand. J. Med. Sci. Sports.

[16] Ferrari G.L.M., Kovalskys I., Fisberg M., Gomez G., Rigotti A., Sanabria L.Y.C., Garcia M.C.Y., Torres R.G.P., Herrera-Cuenca M., Zimberg I.Z. (2020). Comparison of self-report versus accelerometer-measured physical activity and sedentary behaviors and their association with body composition in Latin American countries. PLoS ONE.

[17] Lear S.A., Hu W., Rangarajan S., Gasevic D., Leong D., Iqbal R., Casanova A., Swaminathan S., Anjana R.M., Kumar R. (2017). The effect of physical activity on mortality and cardiovascular disease in 130 000 people from 17 high-income, middle-income, and low-income countries: The PURE study. Lancet.

[18] Koyanagi A., Stubbs B., Vancampfort D. (2018). Correlates of low physical activity across 46 low- and middle-income countries: A cross-sectional analysis of community-based data. Prev. Med..

[19] Tudor-Locke C., Johnson W.D., Katzmarzyk P.T. (2009). Accelerometer-determined steps per day in US adults. Med. Sci. Sports Exerc..

[20] Ferrari G.L., Oliveira L.C., Araujo T.L., Matsudo V., Barreira T.V., Tudor-Locke C., Katzmarzyk P. (2015). Moderate-to-vigorous physical activity and sedentary behavior: Independent associations with body composition variables in brazilian children. Pediatr. Exerc. Sci..

[21] Luis de Moraes Ferrari G., Kovalskys I., Fisberg M., Gomez G., Rigotti A., Sanabria L.Y.C., Garcia M.C.Y., Torres R.G.P., Herrera-Cuenca M., Zimberg I.Z. (2019). Original research Socio-demographic patterning of self-reported physical activity and sitting time in Latin American countries: Findings from ELANS. BMC Public Health.

[22] Yang C.C., Hsu Y.L. (2010). A review of accelerometry-based wearable motion detectors for physical activity monitoring. Sensors.

[23] Dwyer T., Hosmer D., Hosmer T., Venn A.J., Blizzard C.L., Granger R.H., Cochrane J.A., Blair S.N., Shaw J.E., Zimmet P.Z. (2007). The inverse relationship between number of steps per day and obesity in a population-based sample: The AusDiab study. Int. J. Obes..

[24] Mantovani A.M., Duncan S., Codogno J.S., Lima M.C., Fernandes R.A. (2016). Different amounts of physical activity measured by pedometer and the associations with health outcomes in adults. J. Phys. Act. Health.

[25] Fisberg M., Kovalskys I., Gomez G., Rigotti A., Cortes L.Y., Herrera-Cuenca M., Yepez M.C., Pareja R.G., Guajardo V., Zimberg I.Z. (2016). Latin American study of nutrition and health (ELANS): Rationale and study design. BMC Public Health.

[26] Ferrari G.L.M., Kovalskys I., Fisberg M., Gomez G., Rigotti A., Sanabria L.Y.C., Garcia M.C.Y., Torres R.G.P., Herrera-Cuenca M., Zimberg I.Z. (2020). Methodological design for the assessment of physical activity and sedentary time in eight Latin American countries–The ELANS study. MethodsX.

[27] NCHS (1996). Analytic and Reporting Guidelines: The Third National Health and Nutrition Examination Survey, NHANES III (1988–94). https://wwwn.cdc.gov/nchs/data/nhanes3/2a/NH3ECG-acc.pdf.

[28] Batista M.B., Romanzini C.L.P., Barbosa C.C.L., Blasquez Shigaki G., Romanzini M., Ronque E.R.V. (2019). Participation in sports in childhood and adolescence and physical activity in adulthood: A systematic review. J. Sports Sci..

[29] Barriera T.V., Tudor-Locke C., Champagne C.M., Broyles S.T., Johnson W.D., Katzmarzyk P.T. (2013). Comparison of GT3X accelerometer and YAMAX pedometer steps/day in a free-living sample of overweight and obese adults. J. Phys. Act. Health.

[30] Chomistek A.K., Yuan C., Matthews C.E., Troiano R.P., Bowles H.R., Rood J., Barnett J.B., Willett W.C., Rimm E.B., Bassett D.R. (2017). Physical activity assessment with the actigraph GT3X and doubly labeled water. Med. Sci. Sports Exerc..

[31] Brond J.C., Arvidsson D. (2016). Sampling frequency affects the processing of Actigraph raw acceleration data to activity counts. J. Appl. Physiol..

[32] Troiano R.P., Berrigan D., Dodd K.W., Masse L.C., Tilert T., McDowell M. (2008). Physical activity in the United States measured by accelerometer. Med. Sci. Sports Exerc..

[33] Ferrari G.L.M., Kovalskys I., Fisberg M., Gomez G., Rigotti A., Sanabria L.Y.C., Garcia M.C.Y., Torres R.G.P., Herrera-Cuenca M., Zimberg I.Z. (2019). Socio-demographic patterning of objectively measured physical activity and sedentary behaviours in eight Latin American countries: Findings from the ELANS study. Eur. J. Sport Sci..

[34] Lohman T.G., Roche A.F., Martorell R. (1988). Anthropometric Standardization Reference Manual.

[35] World Health Organisation (WHO) (2008). Waist Circumference and Waist–Hip Ratio. Report of a WHO Expert Consultation.

[36] Cornier M.A., Despres J.P., Davis N., Grossniklaus D.A., Klein S., Lamarche B., Lopez-Jimenez F., Rao G., St-Onge M.P., Towfighi A. (2011). Assessing adiposity: A scientific statement from the American Heart Association. Circulation.

[37] IBM Corp (2013). IBM SPSS Statistics for Windows, Version 22.0.

[38] Cohen J. (1998). Statistical Power Analysis for Behavioural Sciences.

[39] Yamamoto N., Miyazaki H., Shimada M., Nakagawa N., Sawada S.S., Nishimuta M., Kimura Y., Kawakami R., Nagayama H., Asai H. (2018). Daily step count and all-cause mortality in a sample of Japanese elderly people: A cohort study. BMC Public Health.

[40] Hall K.S., Hyde E.T., Bassett D.R., Carlson S.A., Carnethon M.R., Ekelund U., Evenson K.R., Galuska D.A., Kraus W.E., Lee I.M. (2020). Systematic review of the prospective association of daily step counts with risk of mortality, cardiovascular disease, and dysglycemia. Int. J. Behav. Nutr. Phys. Act..

[41] Duncan M.J., Minatto G., Wright S.L. (2016). Dose-response between pedometer assessed physical activity, functional fitness, and fatness in healthy adults aged 50–80 years. Am. J. Hum. Biol..

[42] Fogelholm M., Malmberg J., Suni J., Santtila M., Kyrolainen H., Mantysaari M. (2006). Waist circumference and BMI are independently associated with the variation of cardio-respiratory and neuromuscular fitness in young adult men. Int. J. Obes..

[43] Taylor A.E., Ebrahim S., Ben-Shlomo Y., Martin R.M., Whincup P.H., Yarnell J.W., Wannamethee S.G., Lawlor D.A. (2010). Comparison of the associations of body mass index and measures of central adiposity and fat mass with coronary heart disease, diabetes, and all-cause mortality: A study using data from 4 UK cohorts. Am. J. Clin. Nutr..

[44] Tchoukalova Y.D., Koutsari C., Votruba S.B., Tchkonia T., Giorgadze N., Thomou T., Kirkland J.L., Jensen M.D. (2010). Sex- and depot-dependent differences in adipogenesis in normal-weight humans. Obesity.

[45] Jones S.A., Wen F., Herring A.H., Evenson K.R. (2016). Correlates of US adult physical activity and sedentary behavior patterns. J. Sci. Med. Sport.

[46] Van Ballegooijen A.J., Van der Ploeg H.P., Visser M. (2019). Daily sedentary time and physical activity as assessed by accelerometry and their correlates in older adults. Eur. Rev. Aging Phys. Act..

[47] Davis M.G., Fox K.R., Hillsdon M., Sharp D.J., Coulson J.C., Thompson J.L. (2011). Objectively measured physical activity in a diverse sample of older urban UK adults. Med. Sci. Sports Exerc..

[48] Diaz-Martinez X., Garrido A., Martinez M.A., Leiva A.M., Alvarez C., Ramirez-Campillo R., Cristi-Montero C., Rodriguez F., Salas-Bravo C., Duran E. (2017). Correlates of physical inactivity: Findings from the Chilean National Health Survey 2009–2010. Rev. Med. Chil..

[49] Diaz-Martinez X., Steell L., Martinez M.A., Leiva A.M., Salas-Bravo C., Labrana A.M., Duran E., Cristi-Montero C., Livingstone K.M., Garrido-Mendez A. (2018). Higher levels of self-reported sitting time is associated with higher risk of type 2 diabetes independent of physical activity in Chile. J. Public Health.

[50] Cuberek R., Pelclova J., Gaba A., Pechova J., Svozilova Z., Pridalova M., Stefelova N., Hron K. (2019). Adiposity and changes in movement-related behaviors in older adult women in the context of the built environment: A protocol for a prospective cohort study. BMC Public Health.

[51] Dohrn I.M., Kwak L., Oja P., Sjostrom M., Hagstromer M. (2018). Replacing sedentary time with physical activity: A 15-year follow-up of mortality in a national cohort. Clin. Epidemiol..

[52] Rezende L.F.M., Lee D.H., Ferrari G., Giovannucci E. (2020). Confounding due to pre-existing diseases in epidemiologic studies on sedentary behavior and all-cause mortality: A meta-epidemiologic study. Ann. Epidemiol..

[53] Ferrari G.L.M., Victo E.R., Kovalskys I., Mello A.V., Previdelli A.N., Sole D., Fisberg M. (2020). Sedentary behavior, physical activity and body composition in adults. Rev. Assoc. Med. Bras..

[54] Arias Tellez M.J., Acosta F.M., Sanchez-Delgado G., Martinez-Tellez B., Munoz-Hernandez V., Martinez-Avila W.D., Henriksson P., Ruiz J.R. (2020). Association of neck circumference with anthropometric indicators and body composition measured by dxa in young spanish adults. Nutrients.

